# Global distribution of clay-size minerals on land surface for biogeochemical and climatological studies

**DOI:** 10.1038/sdata.2017.103

**Published:** 2017-08-22

**Authors:** Akihiko Ito, Rota Wagai

**Affiliations:** 1 National Institute for Environmental Studies, Tsukuba 305-8506, Japan; 2 Japan Agency for Marine-Earth Science and Technology, Yokohama 236-0001, Japan; 3 National Agriculture and Food Research Organization, Tsukuba 305-8604, Japan

**Keywords:** Element cycles, Mineralogy, Carbon cycle

## Abstract

Clay-size minerals play important roles in terrestrial biogeochemistry and atmospheric physics, but their data have been only partially compiled at global scale. We present a global dataset of clay-size minerals in the topsoil and subsoil at different spatial resolutions. The data of soil clay and its mineralogical composition were gathered through a literature survey and aggregated by soil orders of the Soil Taxonomy for each of the ten groups: gibbsite, kaolinite, illite/mica, smectite, vermiculite, chlorite, iron oxide, quartz, non-crystalline, and others. Using a global soil map, a global dataset of soil clay-size mineral distribution was developed at resolutions of 2' to 2° grid cells. The data uncertainty associated with data variability and assumption was evaluated using a Monte Carlo method, and validity of the clay-size mineral distribution obtained in this study was examined by comparing with other datasets. The global soil clay data offer spatially explicit studies on terrestrial biogeochemical cycles, dust emission to the atmosphere, and other interdisciplinary earth sciences.

## Background & Summary

With an equivalent spherical diameter of<2 μm, clay particles are produced through various geochemical processes such as weathering and metamorphism^1^. Clay exists in the Earth’s crust, ocean sediments, and atmospheric aerosols. Clay is one of the fundamental constituents of soil; it influences soil physical and chemical attributes, soil biogeochemical functions, and the services it provides^2,3^. Because of their size and microstructure, clay-size fine particle fraction (hereafter clay-size minerals) account for major portions of soil specific surface area, allowing interaction with inorganic ions and organic compounds^4^. Clay lends the properties of viscosity and plasticity to wet soils and causes concretion as a result of baking and burning^5^. Interaction of clays with organic matter facilitated by soil microbial activity leads to the formation of characteristic pore-rich structure in soil called aggregates, which enhance aeration and water retention, and stabilize soil organic matter^6,7^. These characteristics are critical for supplying terrestrial plants with water and nutrients for growth and survival. Moreover, clay is a major source of airborne mineral dust, which is mainly contributed to the atmosphere from arid regions. Compared with silt (2–20 μm diameter) and sand (>20 μm diameter), airborne clay dust has a longer residence time in the atmosphere and influences radiation scattering and cloud formation^8^.

Because of their unique properties and importance in many natural processes, clay-size minerals are frequently of interest in soil biogeochemical and climatological studies. Dust emission schemes implemented in aerosol-chemistry models for atmospheric research consider soil particle size distribution^9,10^. Soil sand and clay fractions are used, together with soil bulk density, in many hydrological and biogeochemical models to determine soil water-holding capacity and hydraulic conductivity^11,12^. Moreover, some biogeochemical models account for the influence of clay content on the turnover rate of soil organic matter^13,14^. For the purposes of broad-scale application, spatially explicit datasets of major soil properties including clay content have been constructed^15–17^. However, clay (<2 μm sized particles) encompasses a variety of minerals that differ widely in physical structure and chemical composition and reactivity, and are distributed heterogeneously on earth surface, both horizontally and vertically. Therefore, utility may be found not only in the distribution of total clay content in soil but also in the mineral composition of the clay to account for patterns in soil function or behavior. Because of the technical difficulty in quantifying clay-size minerals^18^, soil datasets published to date usually exclude information on clay-size mineral composition, except for limited geographical regions (e.g., Australia^19^). Several dust emission datasets provide global maps of soil clay-size minerals^20–22^ for dust sources such as the near-surface soils of dry regions. However, these dust source datasets are not useful for hydrological and biogeochemical studies in humid regions where subsoil properties may be required.

We therefore developed a dataset of major soil clay-size minerals covering the global land surface for both topsoil (near-surface) and subsoil at different spatial resolutions. This dataset is intended for application in a variety of earth science fields that require interdisciplinary data. We gathered observational data on clay-size minerals through a literature survey and meta-analysis. Most observations were originally obtained by X-ray diffraction (XRD) analysis. The range of clay-size minerals that occur in soils were classified into ten groups: gibbsite, kaolinite, illite/mica, smectite, vermiculite, chlorite, iron (Fe) oxide, quartz, non-crystalline (amorphous and short-range-order minerals), and others. We then aggregated the clay-size mineral composition data on the basis of 12 soil orders^23^. Using a global map of soil orders and additional soil datasets, we developed global maps of clay-size mineral abundances in topsoil and subsoil at a resolution of 2′ grid cells (about 3.7 km) and, by averaging, at lower spatial resolutions. We examined uncertainties in the dataset by statistical (i.e., Monte Carlo) methods and by comparison with previous datasets.

While the new dataset developed here has room for improvement, it can facilitate continental-scale studies of biogeochemistry and climatology by providing more precise soil properties related to, for example, soil cation exchange capacity and dust emission. The dataset should also find applications in interdisciplinary studies in fields such as hydrology and agronomy, both as input data for model simulations and for the interpretation of observational data.

## Methods

### Meta-analysis

Records of the clay-size mineral composition of terrestrial soils were mainly collected by literature survey using the search engines Web of Science (Thomson Reuters, New York, NY) and Google Scholar (Alphabet Inc., Mountain View, CA). We attempted to gather as much data as possible for all the orders of Soil Taxonomy^23^, a soil classification system that is both practical and genetic as it considers all soil formation factors (i.e., climate, parent material, topography, biota, and time). It also captures the variation in the degree of weathering from immature Entisols and young Inceptisols to highly weathered Ultisols and Oxisols. This soil classification system has been commonly used in soil science and is compatible with the World Reference Base^24^, an international standardized soil classification system. Histosol order was excluded from the present study because it is distributed mainly in peat lands and is primarily composed of organic materials with little mineral matter. For the same reason, data from organic layers (O or A_0_ horizon) above mineral soil layers were not considered. We selected records from papers that contained all the following information for a soil sample: the sampling location, the soil order at the site (or a name from another soil classification system that could be translated to a Soil Taxonomy order), the depth or soil layer sampled, and quantitative data on clay-size mineral composition (e.g., percent of the clay fraction of each constituent, referred as % clay hereafter). Papers that only qualitatively described which clay-size mineral was predominant in the soil sample were not used. In the literature we collected, the majority of measurements of clay-size mineral composition used the XRD analysis method^25,26^. Several studies also adopted other analytical methods to identify and quantify clay-size minerals, such as scanning or transmission electron microscopy, infrared spectroscopy, differential thermal analysis, and thermogravimetric analysis^18^ as well as selective dissolution^27,28^ for the non-crystalline category. When available, other information on the soil samples was extracted for future analyses. These comprised bulk density; sand, silt, and clay composition; cation exchange capacity; specific surface area; and organic carbon content. At present, we gathered 168 records from 27 papers (Data Citation 1), which satisfied the conditions mentioned above, representing all the soil orders and different continental regions.

In the collected literature, clay-size fraction (not only clay minerals but also other substances) was classified using various methods and nomenclatures. To maximize the applicability of the data to biogeochemical studies, we aggregated clay-size minerals into ten groups (Table 1): gibbsite, kaolinite, illite/mica, smectite, vermiculite, chlorite, iron oxide, quartz, non-crystalline, and others. Illite and mica were grouped together, because illite may form from the weathering of potassium-rich mica (muscovite), the most common mineral in mica family. Among 2:1 type layer silicate clay minerals, vermiculite and smectite were separated from illite due to their expandability of their interlayers that lead to much higher chemical reactivity. Smectite-group minerals (e.g., montmorillonite) is particularly expandable and thus separated from vermiculite. Hydroxyl-interlayered-vermiculite (HIV) and hydroxyl-interlayered smectite (SIV), often present in acidic soils, are included under vermiculite category. Iron oxide, hydroxide, and oxyhydroxide are collectively called Fe oxides here. Fe oxide, mainly present as hematite and goethite, is separately considered from other clay-size minerals because this is an important clay-sized mineral group especially in weathered tropical soils and those derived from mafic materials and plays a unique role in plant nutrition and soil microbial metabolism^29^. The non-crystalline category, estimated from selective dissolution data, contained several different amorphous and short-range order minerals including volcanic glass in Andosols and acid oxalate soluble phases of Al and Fe oxides. For each record, total clay-size mineral fraction of 10 groups in Table 1 was normalized to 100%. The others category was adopted as a catch-all for clay-size constituents with a low frequency of occurrence, including feldspar, and mixed-layer clay-size minerals. The classification is not intended to remain fixed and could be revised in response to improved understanding or to meet the requirements of different applications.

**Figure 1:**
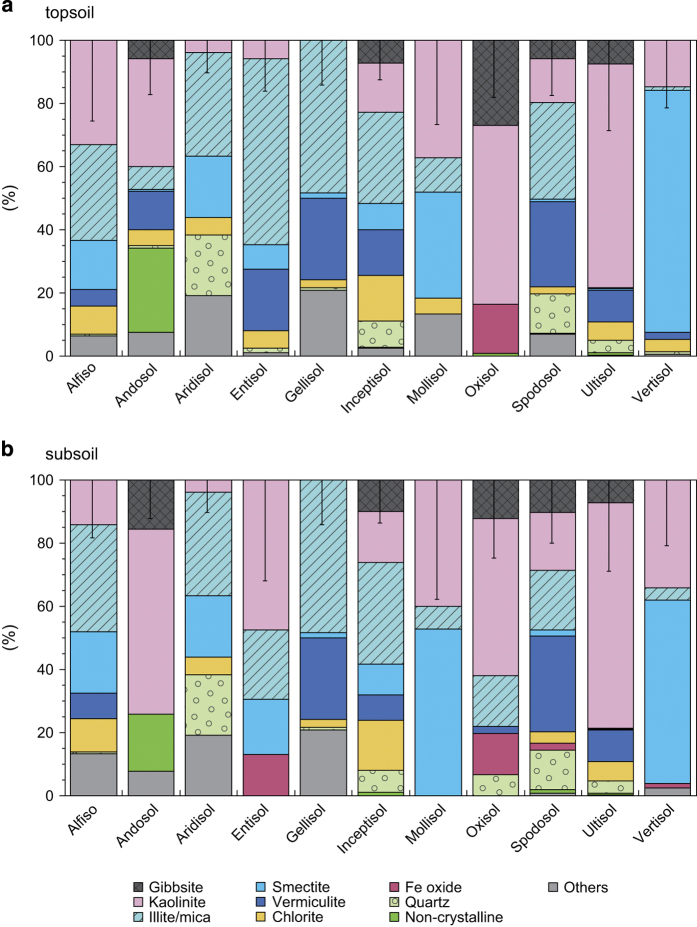
Mean clay-size mineral composition for each of the soil orders of Soil Taxonomy. The data were derived from the literature, and shown for the (**a**) topsoil and (**b**) subsoil, separately. The error bar at the top of each column shows the range of the fraction-weighted standard deviation obtained from data in the literature.

In this study, we adopted a global definition of topsoil as the soil layer from the ground surface to 0.3 m depth when the sampling depth was given, or as soil in the A horizon when the sampling depth was described in terms of soil profile layers. Similarly, subsoil was defined as soil below 0.3 m depth or the B horizon. Soils obtained from below 2 m depth or the C horizon were not recorded, because our focus was primarily on the biogeochemically active soil components. To avoid overrepresentation of a few intensive studies, multiple samples from a single site were averaged by site and soil layer. We then calculated statistical metrics (e.g., mean, median, and standard deviation) of the clay-size mineral fractions for the topsoil and subsoil of each Soil Taxonomy order. The mean clay-size mineral composition for the 11 soil orders obtained from the meta-analysis clearly indicated that clay-size mineral composition differs widely among soil orders (Fig. 1).

### Mapping

Several methods are available to convert georeferenced point data into a continuous domain, such as linear interpolation and geostatistical techniques like kriging. In light of the discontinuous nature of clay composition and the sparsity of the available data, we adopted a simple and robust assignment method on the basis of the soil-type map. We used a global map of Soil Taxonomy orders and suborders produced by the Natural Resources Conservation Service of the United States Department of Agriculture (data obtained from: https://www.nrcs.usda.gov/Internet/FSE_DOCUMENTS/nrcs142p2_052837.zip, last accessed: July 7, 2017) produced by reclassifying the base maps of the FAO/UNESCO Soil Map of the World. The spatial resolution of the soil map is a grid defined by cells of 2' latitude and longitude (about 3.7 km on the equator). The average clay composition (Fig. 1) was assigned to each grid cell on the basis of the dominant soil order for the cell (areas of glaciers and ice sheets, Histosol in wetlands, and shifting desert sands were excluded). To enable the data to be used as inputs for global modeling at spatial resolutions coarser than 2', we also prepared versions of the maps with larger grids (Data Citation 1). The multiple 2' values contained in a larger grid cell would be averaged to produce a value for the larger cells. We produced versions with cell sizes of 5', 15', 30', 1°, and 2°.

The clay composition data mentioned above indicate *F*_*CM*_ (% clay), which is the proportion of the soil’s clay occupied by a given clay-size mineral group, for each of the ten groups. To obtain the absolute amount of a clay-size mineral in the soil column (i.e., weight of mineral per unit area, *W*_*CM*_, kg m^−2^), we used the following equation:

$$W_{CM}=ST\times BD\times (1-F_{GV}/100)\times F_{CL}/100\times F_{CM}/100$$

where *ST* is soil depth or layer thickness (m), *BD* is soil bulk density (kg m^−3^), *F*_*GV*_ is the gravel fraction (%), and *F*_*CL*_ is the soil clay content (%). These additional soil data (*BD*, *F*_*GV*_, and *F*_*CL*_) are available from global datasets such as the Harmonized World Soil Database (HWSD), version 1.21, developed by the FAO and International Institute of Applied System Analysis^16^. The HWSD was compiled from different source soil maps and pedon data to maximize the usability of existing soil data.

### Code availability

The global map datasets were produced using a purpose-built program written in C with the standard mathematical library. The code has been compiled for a variety of platforms (e.g., Windows, UNIX, and MacOS), requires 1.4 to 11 GB memory to run dependent on platform and spatial resolution, and is available from the corresponding author upon request.

## Data Records

The meta-analysis dataset (clay_meta_data_v1.csv; Data Citation 1) includes the following fields for each record:

Record ID

Soil order name

Topsoil (T) or subsoil (S)

*F*_*CM*_ for gibbsite group (% clay)

*F*_*CM*_ for kaolinite group (% clay)

*F*_*CM*_ for illite/mica group (% clay)

*F*_*CM*_ for smectite group (% clay)

*F*_*CM*_ for vermiculite group (% clay)

*F*_*CM*_ for chlorite group (% clay)

*F*_*CM*_ for iron oxide group (% clay)

*F*_*CM*_ for quartz group (% clay)

*F*_*CM*_ for non-crystalline group (% clay)

*F*_*CM*_ for others group (% clay).

References

The global map data file includes the following variables for each grid cell of the topsoil and subsoil files, respectively:

*F*_*CM*_ and *W*_*CM*_ for gibbsite group (% clay or kg m^−2^)

*F*_*CM*_ and *W*_*CM*_ for kaolinite group (% clay or kg m^−2^)

*F*_*CM*_ and *W*_*CM*_ for illite/mica group (% clay or kg m^−2^)

*F*_*CM*_ and *W*_*CM*_ for smectite group (% clay or kg m^−2^)

*F*_*CM*_ and *W*_*CM*_ for vermiculite group (% clay or kg m^−2^)

*F*_*CM*_ and *W*_*CM*_ for chlorite group (% clay or kg m^−2^)

*F*_*CM*_ and *W*_*CM*_ for quartz group (% clay or kg m^−2^)

*F*_*CM*_ and *W*_*CM*_ for iron oxide group (% clay or kg m^−2^)

*F*_*CM*_ and *W*_*CM*_ for non-crystalline group fraction (% clay or kg m^−2^)

*F*_*CM*_ and *W*_*CM*_ for others group (% clay or kg m^−2^)

Dominant (i.e., most abundant) clay-size mineral group

Uncertainty due to sample variability (%)

The files (Data Citation 1) are in NetCDF-4 format, library version 4.3.1.1 (http://www.unidata.ucar.edu/software/netcdf/), and available as clay_XXXX_YY_v1r1.nc4, where XXXX indicates fraction or weight data and YY denotes spatial resolution. Files with different mesh sizes are provided: 2' (YY=2 m), 5' (YY=5 m), 15' (YY=qd), 30' (YY=hd), 1°(YY=1d), and 2°(YY=2d); note that 2’ data were divided into three files due to their large size.

## Technical Validation

### Estimation uncertainty

**Figure 2:**
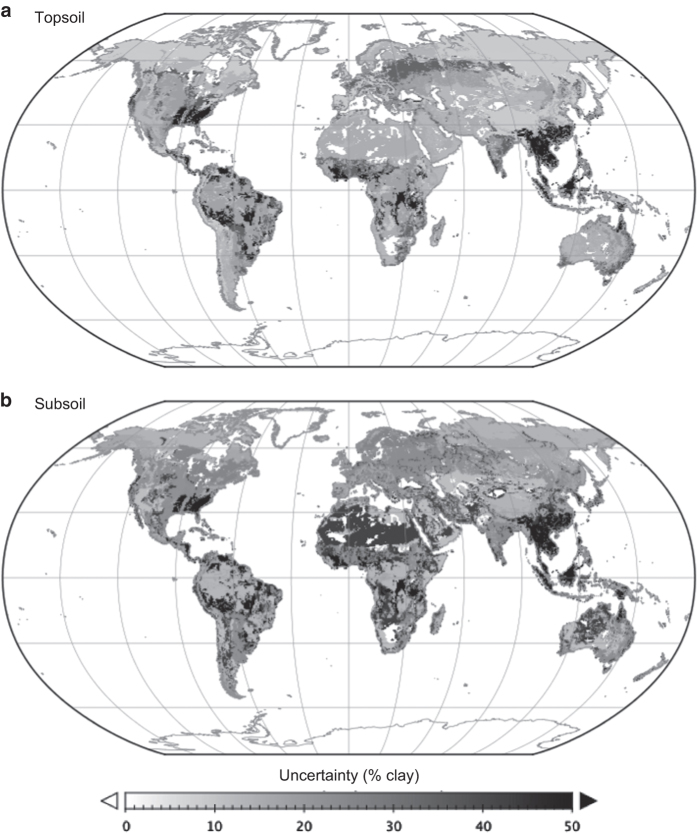
Distribution of combined weighted uncertainty in the estimates of clay-size mineral composition. Data are shown for (**a**) topsoil and (**b**) subsoil.

We evaluated the uncertainty of the calculated clay-size mineral composition values using the Monte Carlo method. The standard deviation values of clay-size mineral composition obtained by the meta-analysis were used to define ranges for random error generation, assuming a Gaussian distribution, to add to the average composition values (Fig. 1). Uncertainty in the clay-size mineral abundance determinations in the source data (e.g., several data were given as abundance level from + to ++++) was incorporated by assigning different values to each category. Namely, we assigned 1–10% to the low abundance level of ‘+’ and 20–60% to the high abundance level of ‘++++’; intermediate values were assigned to ‘++’ and ‘+++’. After assigning the data uncertainty and random error, a total 30,000 sets of global maps were generated and normalized to total 100% as mentioned previously. Note that multiple initial values were used when generating random numbers. Based on the 30,000 combinations of clay-size mineral fractions at each grid, standard deviations were calculated for each clay-size mineral and weighted by the mean clay composition of that mineral before mapping the distribution of uncertainty (Fig. 2).

### Comparison with other datasets

Several clay-size mineral maps have been developed for estimating dust emission^20–22^. The datasets of Clanquin *et al.*^20^ and GMINER30 by Nickovic *et al.*^21^ cover only arid regions such as deserts and dunes, because little dust emission occurs from humid vegetated areas. The present global dataset is consistent with these datasets with respect to major features of clay-size mineral distribution, such as the abundance of illite/mica in arid regions in West Asia and North Africa. The dataset of Clanquin *et al.*^20^ and Nickovic *et al.*^21^ show high quartz content in arid regions in Africa, Asia, and Australia because these datasets also include data for silt. The dataset of Journet *et al.*^22^ covers both arid and humid land areas, except glaciers and wetlands, but only for topsoil subject to aeolian deflation. For dust emission estimation, the dataset separates clay-size fraction into groups for calcite, goethite, feldspar, and hematite, which were aggregated in our dataset. Again, our dataset is consistent with that of Journet *et al.*^22^, in that illite/mica content is high (40–50%) in arid regions and low (<10%) in the humid tropics. The three antecedent datasets focused on dust emission to the atmosphere, ignoring clay functions in terrestrial ecosystems. For example, these datasets do not consider non-crystalline clay, which exerts strong control on soil carbon storage and nutrient cycling^30^. The dataset we have produced provides clay-size mineral composition for both topsoil and subsoil, and for both arid and humid land areas, so unlike the pre-existing datasets it can be used for integrated studies of biogeochemistry and climatology.

### Assessment of the global patterns of clay-size minerals

**Figure 3:**
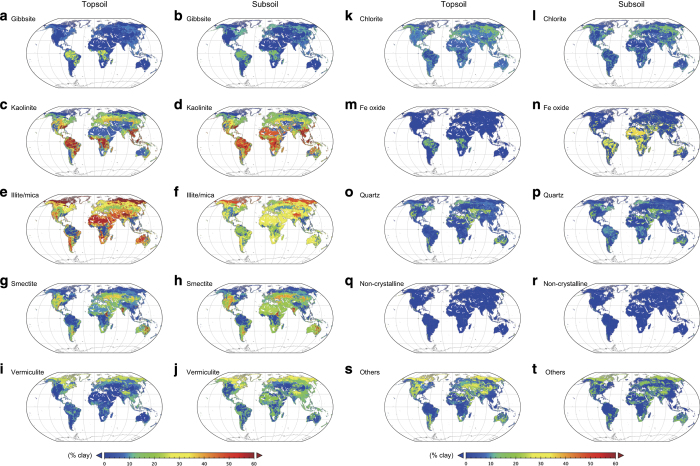
Global terrestrial distribution of clay-size mineral groups as a percent of the soil clay fraction (% clay), excluding areas of ice sheet, glaciers, shifting desert sands and dunes, and wetlands (histosols). (**a**, **b**) Gibbsite, (**c**, **d**) kaolinite, (**e**, **f**) illite/mica, (**g**, **h**) smectite, (**i**, **j**) vermiculite, (**k**, **l**) chlorite, (**m**, **n**) Fe oxide, (**o**, **p**) quartz, (**q**, **r**) non-crystalline, and (**s**, **t**) others. Data for topsoil (**a**, **c**, **e**, **g**, **i**, **k**, **m**, **o**, **q**, **s**) and subsoil (**b**, **d**, **f**, **h**, **j**, **l**, **n**, **p**, **r**, **t**) are shown.

**Figure 4:**
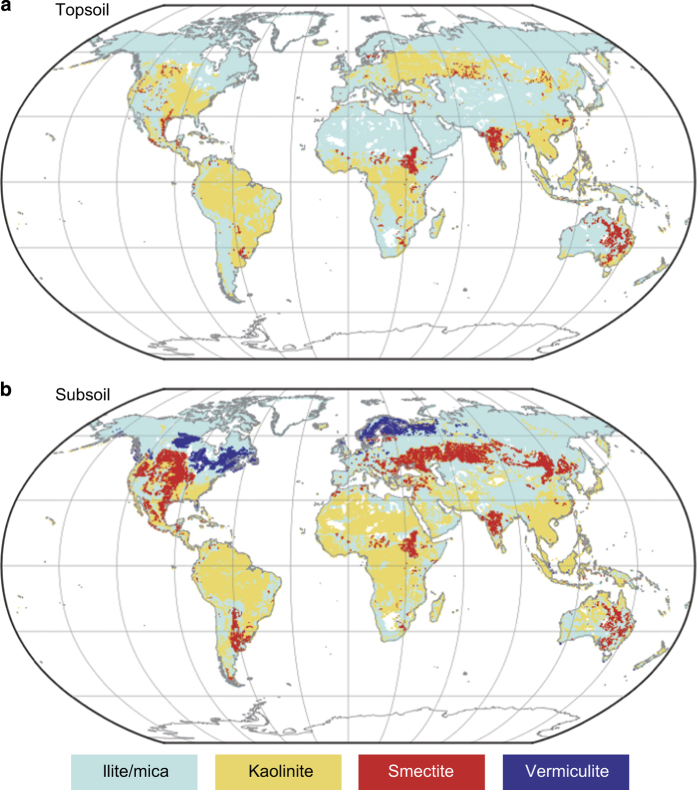
Distribution of the most abundant clay-size mineral group in each grid cell. Data is shown for (**a**) topsoil and (**b**) subsoil. The gibbsite, chlorite, quartz, and non-crystalline groups are not represented because they were never the most abundant.

The global distributions of individual clay-size mineral groups (Fig. 3) and dominant (most abundant) clay-size mineral groups in the topsoil and subsoil (Fig. 4) can be compared with known patterns of distribution. The kaolinite group with 1:1 type layer structure is predominant in the humid tropics, and the illite/mica group is abundant in arid and high-latitude regions. Such distributions are consistent with pedological knowledge about the relationship between weathering intensity and clay-size mineral formation^2,31^. Independent evidence supports the validity of the present dataset, at least for continental-scale patterns. The global pattern of dominant clay-size minerals in ocean sediments^32^ reflects riverine transportation from the land and is consistent with the terrestrial distributions revealed in our dataset (Fig. 4).

**Figure 5:**
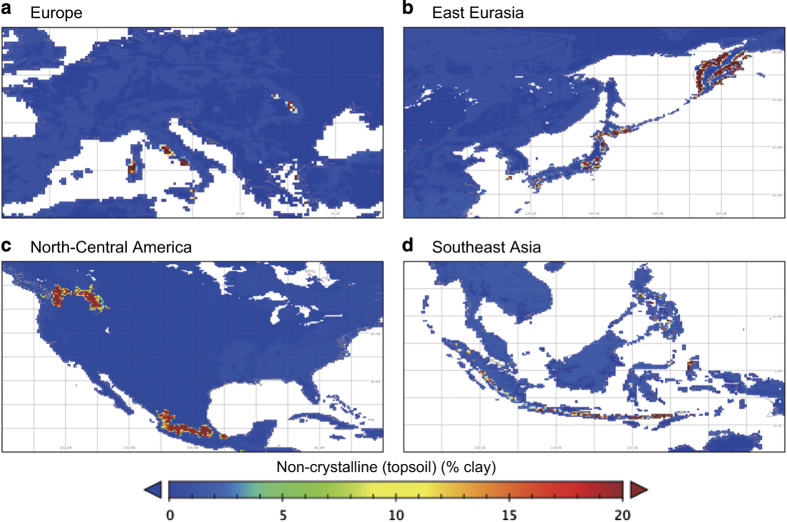
Regional distribution of non-crystalline (principally allophane) clay-size fraction group. Data are shown at 15' resolution for (**a**) Europe, (**b**) East Eurasia, (**c**) North–Central America, and (**d**) Southeast Asia.

Several clay-size minerals are known to have localized distributions, and the dataset appropriately captures their characteristics. For example, Vertisols contain a high proportion of smectite in the topsoil, and both this soil order and the distribution of topsoil smectite in our dataset occur mainly in eastern Australia, central India, and east-central Africa. Allophane and imogolite, major constituents in the non-crystalline group, are formed mainly by the weathering of volcanic parent materials (e.g., tephra), so the non-crystalline clay-size minerals group shows a highly localized distribution in areas associated with geologically recent volcanic activity in East Asia, Southeast Asia, northwestern North America, Central to South America, and southern Europe (Fig. 5). The distributions of these soil clay-size minerals provided by the dataset would be effective in characterizing associated soil functions at each grid cell.

**Figure 6:**
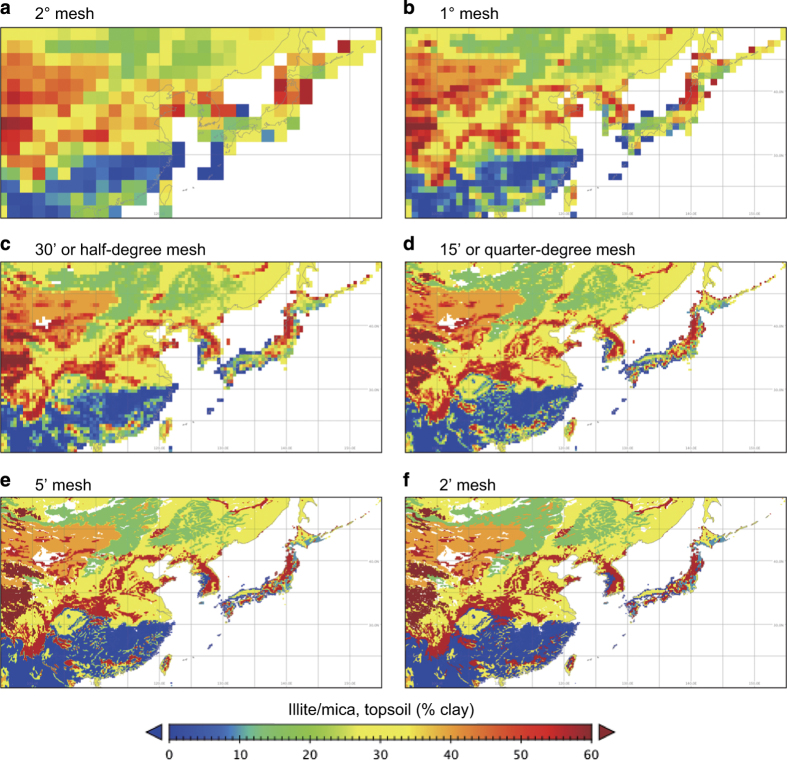
Spatial pattern of clay-size mineral distribution shown at different spatial resolutions. Data of illite/mica clay-size mineral group, as percent of the clay fraction (% clay) of topsoil in East Asia, are shown at spatial resolutions of (**a**) 2°, (**b**) 1°, (**c**) 30', (**d**) 15', (**e**) 5', and (**f**) 2'.

The map data can be displayed at different spatial resolutions (Fig. 6). Apparently, the base map with 2' grid captures spatial heterogeneity in the greatest detail. The low-resolution maps lose fine-scale heterogeneity, but capture broad variations such as the change in clay-size mineral composition with climate gradients and parent materials. The lower resolution data ware produced as a secondary exercise to support global modeling studies.

### Ways to improve dataset

While the quick-look comparison and assessment above showed reasonable results, there are multiple ways to improve the dataset. Our approach was based on (i) the global soil distribution map at soil ‘order’ level in Soil Taxonomy system, and (ii) the assumption of relatively small mineralogical variation within each soil order.

One could further take advantage of the wealth of information present in the soil classification systems used internationally such as World Reference Base for Soil Resources (WRB) and Soil Taxonomy. The WRB system classifies global soils into 32 ‘reference soil groups’ instead of 12 soil ‘orders’ in Soil Taxonomy. While the availability is limited to some geographic regions, ‘soil family’ level information in Soil Taxonomy is directly tied to dominant soil mineral types (e.g., kaolinitic, smectitic, gibbsitic) and particle-size distribution (e.g., clayey, loamy, sandy).

Fe oxide and non-crystalline groups are important mineral phases not adequately represented in the current dataset because the techniques to assess silicate clays (e.g., XRD) are not the most suitable method to characterize Fe oxides and other non-crystalline phases such as allophane. However, the quantitative estimation of these mineral groups is possible by selective dissolution approaches^27,28^. In fact, these are standard techniques in soil science and thus a large literature is present (often without XRD data), which can be incorporated into the dataset.

The assumption of small within-soil-order variation needs to be critically examined to improve the quality of the dataset. Some soil orders are characterized by specific set of mineral types due to the strong influence of unique parent material, climate regime, and weathering intensity (e.g., Andisols, Vertisols, and Oxisols). On the other hand, less developed soil orders such as Inceptisols and Entisols are likely to show much higher variation in clay-size mineral composition depending on soil formation factors (especially parent material, topography, climate). Furthermore, human activity such as deforestation, land-use change, and tillage could significantly degrade soils and change mineralogical composition of clay-size fraction within each soil order, for instance, by erosion-induced displacement^3^.

The present dataset is, therefore, a first step towards a complete global distribution map for clay-size minerals and relevant soil properties. Nevertheless, the current dataset has some values to facilitate ongoing biogeochemical studies and we can make improvements as discussed above.

## Usage Notes

The meta-analysis dataset can be used to determine clay-size mineral composition for the soil orders of Soil Taxonomy and corresponding soil categories from other classifications. The map data can be used to specify the regional distribution of characteristic clay-size constituents (such as smectite and non-crystalline mineral including allophane) affecting phenomena of interest in the fields of geomorphology and plant productivity. For global studies, the dataset can be used to characterize soil properties such as water-holding capacity, cation exchange capacity, and dust emissivity for studies in the fields of biogeochemistry and climatology. The dataset would be useful for planning soil management in land systems or regions subject to human pressure^33,34^. For example, the current dataset could be used to improve our estimation of soils capacity to form stable aggregate against erosion^35^ and to store C as soil organic matter^36,37^. For example, a soil stability index could be developed on the basis of the clay mineralogy and relevant properties data.

## Additional Information

**How to cite this article:** Ito, A. & Wagai, R. Global distribution of clay-size minerals on land surface for biogeochemical and climatological studies. *Sci. Data* 4:170103 doi: 10.1038/sdata.2017.103 (2017).

**Publisher’s note:** Springer Nature remains neutral with regard to jurisdictional claims in published maps and institutional affiliations.

## Supplementary Material



## Figures and Tables

**Table 1:** Summary of clay-size mineral groups.

**Groups**	**Major clay-size minerals**	Structure*
Gibbsite	gibbsite	1 (octahedral)
Kaolinite	kaolinite, halloysite	1:1
Illite/mica	illite, mica	2:1
Smectite	montmorillonite	2:1
Vermiculite	vermiculite	2:1
Chlorite	chlorite	2:1:1
Iron (Fe) oxide	goethite, hematite	mixed
Quartz	quartz	crystal
Non-crystalline	allophane, imogolite, ferrihydrite	amorphous
Others	feldspar, etc.	mixed

*Silicate layer or other structural characteristics of clay-size minerals. Numbers indicate the numbers of tetrahedral: octahedral (: inter-layer) sheets.
